# Barriers and facilitators to accessing urologic cancer treatment for people experiencing homelessness: a systematic review

**DOI:** 10.1097/MOU.0000000000001383

**Published:** 2026-03-18

**Authors:** Sabrina Pfluger, Tobias Fragner, Honja Hama, Igor Grabovac

**Affiliations:** a Department of Social and Preventive Medicine, Center of Public Health, Medical University of Vienna; b Comprehensive Cancer Center, Medical University of Vienna and Vienna General Hospital, Vienna, Austria

**Keywords:** cancer care disparities, homelessness, treatment inequity, urologic oncology

## Abstract

**Purpose of review:**

People experiencing homelessness (PEH) remain an understudied and underserved population in cancer care, particularly regarding access to urologic cancer treatment. This review aims to examine barriers and facilitators PEH encounter when accessing treatment for urologic malignancies.

**Recent findings:**

PEH are found to have increased odds of delayed or disrupted urologic oncological treatment and experience deviations from guideline-based therapy. Problems arise in treatment coordination and organisation, with communication and logistical challenges posing major barriers. Comorbidities and disadvantaged insurance status further exacerbate disparities. The lack of documented reasons for treatment interruptions limits the ability to clarify individual patient needs. Person-centred therapy, housing provision, and additional resources act as facilitators.

**Summary:**

Among the five included studies, two assessed treatment adherence, one evaluated inpatient care delivery for hospitalised PEH, one explored links between housing status, diagnostic stage, and mortality after diagnosis, and one examined time-to-treatment. Present findings indicate an association between housing instability and insufficient care along the urologic cancer pathway. Delayed or distorted therapy, persisting bias and stigma, and personal problems contribute to inequities in the received care. Proposed facilitators range from housing interventions, multidisciplinary and person-led care, to treatment adaptations and participatory approaches in therapy and future research.

## INTRODUCTION

In 2022, more than 2.6 million new cases of urologic cancers were detected worldwide, accounting for 13.1% of all newly reported cancer diagnoses [1]. While these numbers contribute substantially to the global cancer burden, they pose a particular strain to underserved and marginalised individuals, such as people experiencing homelessness (PEH).

PEH are at an increased risk of developing malignancies owing to more frequent exposure to carcinogenic substances, such as tobacco and alcohol [2], as well as social exclusion, poor nutrition [3], and lower levels of cancer-related health literacy [4]. The increased cancer incidence within this community is significantly influenced by limited access to cancer prevention and primary care [2,5], which contributes to low rates of prostate cancer screening and disparities in urologic health for PEH [6,7]. These barriers to care not only impede early detection of malignancies but are also associated with delayed treatment initiation and suboptimal care. The absence of stable housing can impact PEH across multiple dimensions, with negative consequences not only detected in cancer care delivery but also in its outcomes [8]. As a result, housing instability makes a sustainable contribution to oncologic diseases, being among the leading causes of mortality among PEH [9,10].

Historical abuses of PEH in urologic cancer research highlight the long-standing marginalisation of PEH in this field. During the 1950 s and 1960 s, men experiencing homelessness were recruited in Perry Hudson's Skid Row prostate cancer trials, often without adequate information or informed consent to undergo invasive biopsies and radical treatments such as prostatectomy [11]. While numerous studies have examined access to cancer prevention and screening among PEH, the treatment of malignancies and, particularly, the associated challenges and disparities remain largely unexplored. This systematic review addresses this gap by describing the complex barriers and facilitators that PEH encounter across the treatment stages of the urologic cancer pathway. 

**Box 1:**
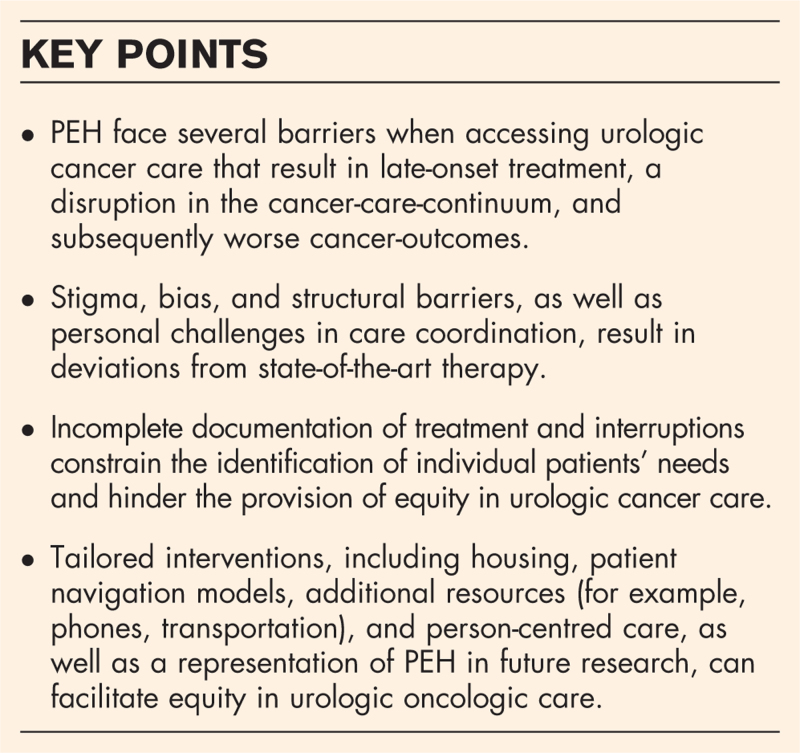
no caption available

## MATERIALS AND METHODS

A systematic review was selected to synthesise evidence on barriers and facilitators to accessing urologic cancer care.

### Eligibility criteria

Studies were included if they represented original research employing a qualitative, quantitative, or mixed-methods approach. Nonoriginal research articles were excluded. Additionally, studies focusing on cancer screening, diagnosis, or palliative care, as well as studies that did not include urologic malignancies in their samples were excluded.

### Literature research

**FIGURE 1:**
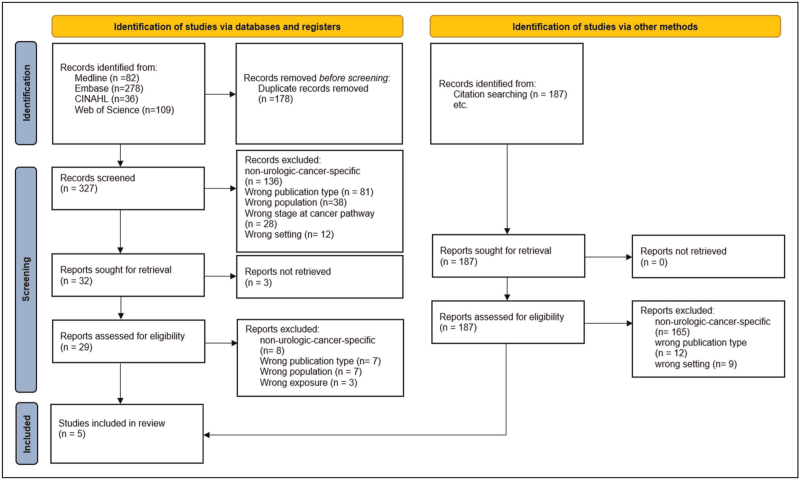
PRISMA flowchart illustrating the literature search process.

The research strategy was developed in collaboration with two research librarians from the Medical University of Vienna. Key search concepts included urologic malignancies, housing status and homelessness, and access to oncologic treatment and related barriers. The initial search was conducted on MEDLINE (PubMed) and Embase (Elsevier). Using the titles and abstracts of the primarily identified articles, the search strategy was refined and extended to CINAHL (Ebsco) and Web of Science (Clarivate). Additionally, eligible studies were identified through citation chaining of relevant literature. The initial search was conducted on August 1, 2025, and an adjusted search was performed on August 11, 2025. The study selection process is illustrated in the PRISMA flowchart (Fig. 1).

### Study and source of evidence selection

Search results were imported into the web application Rayyan [12], where duplicates were removed manually. One researcher (S.P.) and an external facilitator independently screened the studies according to the predefined eligibility criteria. Discrepancies were resolved by discussion, and a consensus on six studies was reached.

### Data extraction and synthesis

Data extraction was performed by one researcher (S.P.) and included author, year, country, study setting, eligibility criteria, sample size, study design, definition of homelessness, and reported barriers and facilitators. The extraction table was then reviewed and discussed in detail with three other researchers (H.H., I.G., T.F.) to assess the eligibility of the full texts. During this process, one listed study was identified as not meeting the criteria; therefore, it was excluded, yielding a final sample of five studies. Key results were initially identified through full-text reading. Subsequently, the extracted data were organised into barriers and facilitators, followed by a categorisation and clustering of the reported factors. The analysis produced an overview of the key factors influencing PEHs’ access to urologic cancer care.

## RESULTS

Five studies relevant to our investigation were included in the review. The identified literature was published between 2021 and 2025, as earlier studies provided crucial findings for the field under investigation. The included studies examined associations between housing status and inpatient care delivery among hospitalised patients [13^▪▪^], associations between housing status, stage at diagnosis, and all-cause survival following diagnosis [14^▪^], delays in cancer care initiation [15], and treatment adherence [16,17]. All publications were conducted in the United States, and homelessness was defined heterogeneously across studies, including ICD-10 Z codes (Z59), electronic medical record documentation, public health and county administrative data, patient-reported housing status, and criteria from the U.S. Department of Housing and Urban Development (HUD). As the identification of evidence that exclusively investigates PEH diagnosed with urologic cancer was not feasible, the samples of the included studies also comprise participants with other types of malignancies. Further details of the studies are provided in Table 1.

**Table 1:** Data extraction and study characteristics

Study	Title	Barriers	Facilitators
Decker *et al.* 2024 [14^▪^]	Association of housing status and cancer diagnosis, care coordination, and outcomes in a public hospital: a retrospective cohort study	Limited access to guideline-based care; presentation at advanced stage; poorly addressed risk factors for cancer	Housing first-approach to improve the cancer outcomes; consideration of biography to detect vulnerabilities that impact health and care access; enhanced case management and further investigation in posthospitalisation housing services
Facer *et al.* 2021 [17]	Radiation Therapy Adherence Among Patients Experiencing Homelessness	Barriers consisting of: late onset diagnosis, delayed treatment initiation and high rates of missed RT oncology visits along the treatment pathway; reasons for course-incompletion due to transportation challenges, hospitalisation, drug overdose, inconsistent documentation of reasons for missed appointments (only 48%); nonadherence rate distorted due to outliers; barriers need further investigation and understanding	Consideration of shorter RT courses in case of similar efficacy (<10); attention to outliers in analyses and targeted interventions for outliers gain better adherence across the whole PEH population; documentation of reasons for missed appointments to recognise specific needs; bias- and stigma prevention
Haemmerle *et al.* 2025 [15]	Homelessness and Cancer: A Multi-Site, Mixed Methods Study of Delayed or No Cancer Therapy	Cancer treatment delays were the result of difficulties in communications between the person receiving care and the healthcare team, challenges in patient participation and medical and related challenges to the provision of cancer care; challenges in phone-contact due to lack of cell phones; coexisting psychiatric diagnoses; delayed start of cancer treatment for the majority of patients; challenges in receiving state-of-the-art-cancer-therapy; nonmedical reasons for therapy delays.	Provision of cheap phones to enable contact between PEH and healthcare providers; targeted concurrent therapy in case of psychiatric co-morbidities
Kilic *et al.* 2023 [16]	Cancer Diagnoses and Use of Radiation Therapy Among Persons Experiencing Homelessness	Numerous diagnoses at an advanced or metastatic stage; lack of health insurance among many patients; prescription rate of RT was low; PEH having limited access to financial, logistical, or social resources, resulting in underuse of RT; deviations from the state-of-the-art-therapy; treatment discontinuation reasons not documented completely	Increase access to health insurance, preventive health maintenance, and routine screening; provision of targeted interventions relevant to all physicians
Shah *et al.* 2024 [13^▪▪^]	Inpatient Care and Outcomes Among People With Cancer Experiencing Homelessness	Deviations from guideline-based therapy: PEH are less likely to receive invasive procedures or systemic treatment; high numbers of discharges against medical advice, as well as many unmet needs; more frequent diagnosis of more aggressive cancer histologies may limit treatment options	Short-course radiation, as this can be initiated even during hospitalisation and does not require intensive follow-up, meeting patients’ essential needs

PEH, people experiencing homelessness; RT, radiotherapy.

## BARRIERS

Barriers to accessing cancer care consist of systemic, personal, and organisational factors with many of the barriers arising early in the cancer pathway. Consequently, many cancers are detected at stage 3 or 4, and the presentation of malignancies in a metastatic stage occurs commonly [14^▪^,^16^,^17^]. An investigation by Haemmerle *et al.*[15] illuminates the timely receipt of urologic oncological care. The authors describe delays in the initiation of cancer therapy due to late diagnosis. Another reason for treatment delays was found in missed appointments [17], consistent with Kilic and colleagues’ findings, who reported no significant difference in time to treatment for PEH compared with US-nationwide treatment initiation times, but 58% of patients never achieving a disease-free status [16]. When accessing the association between housing status, stage at diagnosis, and all-cause survival following diagnosis, Decker *et al.*[14^▪^] report a 50% increased hazard of death for patients experiencing homelessness diagnosed at stage four disease.

Four studies identified difficulties in clinical decision-making and deviations from guideline-based therapies among the far-reaching and multidimensional factors contributing to disparities [13^▪▪^,^15^–^17^]. This is reflected in the findings that PEH predominantly receive radiotherapy [13^▪▪^], with particularly high prescription of hypofractionated radiotherapy courses [16,17]. In work by Shah *et al*. [13^▪▪^], an evaluation of the association between housing status and inpatient care delivery, the authors found PEH having lower odds of undergoing invasive procedures or receiving systemic therapy, alongside a higher reported prevalence of more aggressive cancer histologies and more extended hospital stays. Furthermore, their work identified that the number of discharges against medical advice was four times higher among the PEH population [13^▪▪^]. In addition to these systemic barriers, two studies report high rates of no insurance or less-advantaged insurance coverage among PEH [13^▪▪^,^16^].

When assessing treatment adherence, Kilic *et al.*[16] report a lower treatment completion rate among PEH receiving multifraction treatment, and Facer *et al.*[17] report more missed appointments and treatment disruptions in this population. The underlying reasons for missed appointments were documented only partially, in only 48% of cases [17]. Incomplete documentation of treatment interruptions in care-providing institutions has been identified as a major obstacle to clarifying patient needs and to understanding the underlying causes of disruption [16,17].

The identified barriers are far-reaching and also affect treatment administration. Difficulties in care coordination have been reported to arise from organisational challenges, such as limited access to phones or other means of communication, which hinder patient outreach and the exchange of treatment-relevant information [15,16]. Another significant logistical barrier is restricted access to transportation [15–17]. Challenges in maintaining a continuity of care and follow-up are described as associated with persisting somatic and psychiatric comorbidities [13^▪▪^,^15^,^17^].

## FACILITATORS

Although the studies’ authors identified numerous multilevel facilitators, they did not empirically evaluate them. Nevertheless, stable housing emerged across several studies as a fundamental intervention associated with improved access to cancer care, benefits for PEHs’ health outcomes, and a reduction in the competing priorities inherent to homelessness [13^▪▪^,14^▪^,^16^].

Further recommendations from the studies include a multidisciplinary treatment approach and healthcare teams comprising various professions [13^▪▪^,^16^]. Implementing case management supported by social workers and patient navigators, is recommended to overcome barriers related to navigation and coordination within complex healthcare systems [13^▪▪^,14^▪^,^16^].

To address the administrative barriers, Haemmerle *et al.*[15] suggest providing low-cost burner phones to improve communication and coordination between healthcare professionals and PEH. Follow-up appointments via telemedicine, if applicable, as well as the implementation of flexible and extended appointment times and transportation services, are further suggested to mitigate logistical barriers [16]. Moreover, two studies emphasise the importance of maintaining an upright insurance status to ease treatment barriers and, alongside this, the provision of access to preventive and screening programs [14^▪^,^16^].

Following a person-centred approach is frequently described to act as a facilitator [13^▪▪^,14^▪^,^17^]. Included suggestions involve awareness of patients’ biographies and essential needs, with appropriate adaptation of therapy [13^▪▪^,14^▪^]. The identification of factors contributing to missed appointments, accurate documentation of the underlying reasons, and the corresponding implementation of preventive interventions and additional resources [17] is further mentioned. Two studies highlight the co-treatment of persistent comorbidities [15,16] as barrier-easing. Facer and colleagues’ [17] findings – the similar treatment adherence between PEH and housed people to radiotherapy courses with 10 or fewer fractions – can be seen as a facilitator. Apart from PEHs’ ability to complete treatment plans despite numerous barriers, authors also highlight the community's resilience [17].

Three studies propose hypofractionated radiotherapy when oncologically applicable. It is hypothesised that treatment adherence can be improved, as short-course radiotherapy minimises the number of on-site visits, lowers the financial burden of treatment, and allows the start and end of radiotherapy to occur during a single admission without requiring intensive follow-up [13^▪▪^,^16^,^17^].

## DISCUSSION

This review outlines barriers and facilitators affecting access to urologic cancer care for PEH. Disparities arise across the cancer care continuum, including diagnosis-related challenges, disruptions in continuity of care, and deviations in clinical decision-making, reflecting systemic, personal, and administrative barriers.

Due to unstable living conditions, PEH may be forced to prioritise basic needs such as food, water, and shelter over healthcare. This hypothesis is consistent with current evidence indicating that competing priorities negatively affect healthcare engagement among PEH [18,19]. Such prioritisation may explain the underutilisation of screening services and delayed cancer diagnosis. The provision of stable housing, proposed to act as a facilitator, is reported in our results. Alongside this, current evidence indicates that the provision of stable housing is associated with improved care continuity and greater access to resources and cancer screening services among PEH [8,20,21]. These findings suggest that the implementation of Housing First programmes has the potential to successfully address the exclusion of communities experiencing homelessness from cancer care services and to improve health outcomes for PEH. Several blind spots identified throughout the research process may limit understanding of persistent inequities and are likely to contribute to PEHs’ poorer cancer outcomes compared to the housed population. Incomplete data in electronic medical record documentation and public health reporting systems, together with an underrepresentation of PEH in urologic-oncologic research, foster a limited understanding of the underlying reasons for disparities. Notably, none of the included studies investigated the relation between persisting stigma and bias towards unhoused individuals among healthcare professionals, nor the consequences for the cancer care received. Nevertheless, a link seems plausible and may be reflected in the high discharges against medical advice [13^▪▪^,^18^] and in the deviations in clinical decision-making described [13^▪▪^,^15^–^17^]. Poor interactions with healthcare professionals, therefore, can be seen as a major barrier to PEH seeking timely cancer care, possibly affecting individuals’ well being negatively. Experiences of discrimination are reported to lead to mistrust within this underserved community and reinforce the unwillingness to engage with the healthcare system [22,23]. PEH further describe being treated inhumanely by healthcare providers, leaving them with feelings of loneliness and isolation, which are ultimately associated with lower self-worth and self-stigmatisation [23]. By acknowledging positive patient experiences and acting as facilitators, healthcare providers play a pivotal role in improving cancer care for underserved communities. Positive and compassionate encounters and interpersonal aspects of care are crucial, as PEH emphasise the importance of supportive relationships within healthcare settings and the value of healthcare professionals who treat patients in a welcoming and approachable way [18,22,24]. Professionals can create a caring environment by taking time to listen [22,24], remembering details about individuals’ lives [22], and building trustful relationships [18,25]. In light of evidence that person-centred treatment approaches can act as facilitators [13^▪▪^,14^▪^,^17^], the necessity of unbiased attitudes towards highly vulnerable communities needs to be further emphasised. Participatory incorporation of PEH throughout the treatment process, as well as tailored interventions after identifying patients’ individual needs, personal resources, and barriers [22,26], can foster an empowering environment.

Logistical problems are identified to exacerbate inequities in urologic cancer care further and represent a major barrier. Beyond providing transportation services and enhancing communication, current evidence suggests that tailored, multidimensional interventions and strategies are needed to meet patients where they are. These include outreach programs, shelters staffed with medical professionals, expanded oncology teams, and, beyond that, community-based health interventions [26–28,29^▪▪^]. Homelessness is a complex, multifactorial determinant of health. Because the underlying reasons for disparities in urologic cancer care remain poorly understood due to incomplete data in public health reporting systems, it is essential to document causes for missed appointments responsibly to contribute to a better understanding of the underlying causes. Including PEH in urologic oncology trials and following a participatory approach is crucial to ensure that future research adequately captures the challenges and needs of this high-risk population [30]. Reducing disparities in cancer care and improving urologic oncologic outcomes is a transformative process that requires the combined efforts of policymakers, healthcare systems, and professionals, with ongoing consideration of and participation by affected individuals and their lived experiences.

## LIMITATIONS

This review has several limitations. All the included studies were performed in the United States, which limits the generalisability of their findings. The study samples were highly heterogeneous, ranging from multisite to single-institution settings. As the current literature focuses on assessing barriers to access, the identified facilitators are primarily based on the authors’ recommendations presented in the discussion section. Due to no studies focusing solely on urologic cancers in PEH, this review expanded its scope to include studies reporting on other cancer types, which may skew some of the findings. Despite conducting a comprehensive search strategy across five databases, there remains a possibility that relevant studies were not captured. Another limitation arises from the inconsistent definitions of homelessness and the variety of data sources used across the studies. The exclusion of studies published in any language other than English may have resulted in an incomplete capture of relevant evidence.

## CONCLUSION

This review highlights disparities in access to urologic oncologic healthcare for PEH and the resulting negative impact on cancer outcomes. Barriers occur across the whole cancer care continuum, including late onset diagnosis, deviations in prescription of guideline-based therapy, personal and intercurrent medical problems, accompanied by challenges in care coordination and communication. Overcoming these barriers requires person-centred treatment, stable housing, and additional resources, alongside direct, low-threshold healthcare provision. Consistent documentation of missed appointments enhances understanding the underlying factors disrupting cancer care. By addressing stigma and bias, healthcare professionals can contribute to trustful and supportive relationships, create a caring environment, and thereby ensure PEH experiencing positive healthcare encounters.

## Acknowledgements

*The authors thank Birgit Heller and Caroline Reitbrecht from the University Library of the Medical University of Vienna for codeveloping and applying our search strategy. Furthermore, they thank Ramona Afzali for contributing to the screening process and for providing valuable feedback.*

### Financial support and sponsorship

*None*.

### Conflicts of interest

*There are no conflicts of interest.*

## REFERENCES AND RECOMMENDED READING

Papers of particular interest, published within the annual period of review, have been highlighted as:

▪ of special interest
▪▪ of outstanding interest
